# Tuning the Activity of 1,5-Diamino-naphthalene Through an Asymmetric Mono-Amidation with Pyroglutamic Acid

**DOI:** 10.3390/molecules30081802

**Published:** 2025-04-17

**Authors:** Davide Carboni, Marta Cadeddu, Federico Olia, Federico Fiori, Roberto Anedda, Massimo Carraro, Luca Malfatti, Plinio Innocenzi

**Affiliations:** 1 Laboratory of Materials Science and Nanotechnology, CR-INSTM, Department of Biomedical Sciences, University of Sassari, Viale San Pietro 43/B, 07100 Sassari, Italy; 2 Porto Conte Ricerche, Strada Provinciale 55, Porto Conte Capo Caccia, Km. 8400, 07041 Alghero, Italy; 3 Dipartimento di Scienze Chimiche, Fisiche, Matematiche e Naturali, Università degli Studi di Sassari, Via Vienna 2, 07100 Sassari, Italy

**Keywords:** 1,5-diamino-naphthalene, l-pyroglutamic acid, ICG, DPPH, antioxidant, oxidant, fluorescence

## Abstract

The class of diamino-naphthalene exhibits antioxidant properties, which are partly related to the relative positions of the two amino groups. This study demonstrates how the reactivity of one of these compounds, 1,5-diamino-naphthalene (DAN), can be adjusted by introducing a single amide bond through a simple thermal coupling with l-pyroglutamic acid (PyroGlu). The solventless thermal reaction between PyroGlu and DAN at 160 °C yielded a new mono-pyroglutanilide compound (PyroDAN) that was characterized using various analytical techniques, including a thermal and infrared analysis, HRMS (ESI), and one- (1D) and two-dimensional (2D) NMR. The optical properties were investigated using UV-Vis and fluorescence spectroscopy. Additionally, two chemical standard assays were used to measure both the antioxidant and pro-oxidant properties of PyroDAN. The molecule has shown nearly negligible pro-oxidant activity, while a mild antioxidant activity is still retained. These findings indicate that the transformation of DAN into a mono-pyroglutanilide derivative breaks the original molecular symmetry and effectively modifies the electronic distribution of the aromatic system, suppressing the oxidant properties while keeping a mild antioxidant activity. Furthermore, the tuneable fluorescent properties of PyroDAN—the mild antioxidant activity and the inhibition of the cytologically harmful pro-oxidant properties—suggest promising applications in bioimaging and other biological fields.

## 1. Introduction

**Scheme 1:**
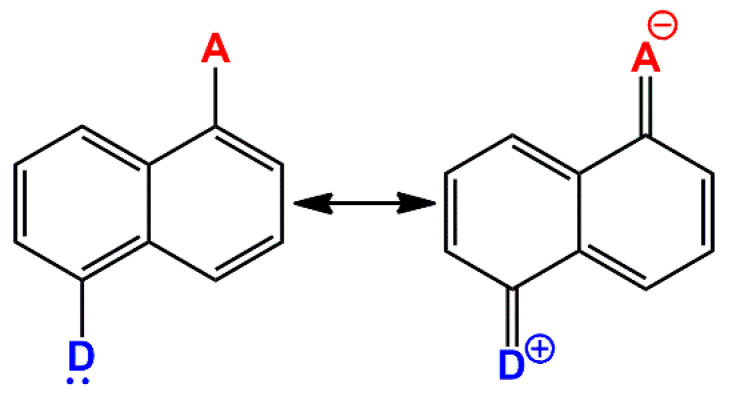
Example of through-conjugation in D-π-A 1,5-disubstituted-naphthalene.

Naphthalene, the smallest member among the polycyclic aromatic hydrocarbons (PAHs) [1], is characterized by two fused benzene rings that form a backbone structure resembling an extended π-conjugated “pipeline”. This molecular structure can be exploited to connect two or more substituents that control the properties and, thus, the reactivity of the naphthalene derivatives. The electron densities of the substituents, as well as their relative positions, play a crucial role, as they can function either as π-donors (D) or π-acceptors (A). This leads to several possible combinations, including D-π-D, A-π-A, and D-π-A, where electrons flow from the donor to the acceptor through the aromatic backbone (*through-conjugation*) [2]. In the case of the D-π-A configuration, this arrangement results in a push–pull (*captodative*) system. The molecular structure of this system can be envisioned as a linear combination of the neutral and charge-separated resonance forms. The following Scheme 1 reports, as an example, these two resonance structures in the case of 1,5-disubstituted-naphthalene.

Due to the significantly lower aromaticity of naphthalene compared with that of benzene, disubstituted-naphthalenes have attracted attention for designing various π-extended structures. For example, some naphthalene compounds with highly conjugated hydroxyl (OH) groups, such as naphthalene diols, have demonstrated promising applications in medical treatments due to their antioxidant, antibacterial, and anti-inflammatory properties [3]. These beneficial effects have been linked to their ability to participate in reactions involving hydrogen atom transfer (HAT) with 2,2-diphenyl-1-picrylhydrazyl (DPPH•) radicals, which are commonly used to assess antioxidant activity. However, the biological properties of these compounds are not limited to naphthalene hydroxyl derivatives; amino-naphthol and amino-naphthalene compounds have also shown particularly effective antioxidant activity [4].

The relative positions of hydroxy and amino substituents on the disubstituted-naphthalene aromatic backbone play a crucial role in determining the radical scavenging activity. This relationship is closely linked to the low bond dissociation energies (BDEs) of the N-H and O-H bonds found in these substituents. Compounds such as dihydroxy-naphthalene, amino-naphthol, and diamino-naphthalene exhibit these characteristics, making them effective hydrogen donors capable of neutralizing free radicals [4]. This activity is correlated with the electron density of the overall molecule, and it can be further influenced by modifying the main functional groups (aniline and/or phenol) with substituents that enhance either the electro-donating or electro-withdrawing characteristics. In this context, Vullev and colleagues investigated how different amide derivatives affected the electronic properties of pyrene [5]. They concluded that the significant electric dipoles and the ability to form ordered hydrogen-bonded networks in the amides, combined with the proper orientation of the amide groups, influence the π-conjugation of the chromophore. This, in turn, affects properties such as optical absorption and reduction potential. Additionally, the amide groups have permanent electric dipoles that influence the electrochemical state of the molecules. These dipoles impact the stabilization of any radical ions that may form, as well as their optical properties [6].

One of the most studied disubstituted-naphthalenes is 1,5-diamino-naphthalene (DAN), a D-π-D type PAH characterized by a higher degree of molecular symmetry. The amino groups exhibit two opposing behaviors: on one hand, the nitrogen atom exerts an inductive electron-withdrawing effect; on the other hand, it displays a strong π-charge resonance, donating its lone pair to the aromatic ring. Typically, the resonance effect predominates, enhancing the negative potentials present in the π regions of the PAH [7]. The symmetric arrangement of the two electro-donating amino groups in DAN appears to be primarily responsible for its strong antioxidant properties [4]. Moreover, in our recent work, we evaluated also the pro-oxidant activity of a DAN derivative obtained by reacting it with the amino acid l-glycine [8]. This reaction produced carbon dot structures that exhibited biocidal properties. In that occasion, we observed that both amino groups of DAN reacted symmetrically with two molecules of l-glycine, resulting in a new compound that retained the original molecular symmetry and most of the properties of DAN. For this reason, we deemed it of interest to understand how the pro-oxidant and antioxidant properties of DAN could be modulated by simultaneously breaking the molecular symmetry and altering the electro-donating features through a simple molecular functionalization.

The present study aimed to investigate the possibility of tuning the oxidant/antioxidant activities of a disubstituted-naphthalene by transforming its structure from a symmetric D-π-D type into an asymmetric captodative system, such as D-π-A. DAN was thus chosen as a model compound primarily because of its highly symmetrical disubstituted structure and the large literature support on its properties. A simple approach to induce this transformation, without altering the π-conjugated backbone of the naphthalene too much, can be achieved; for instance, by reducing the electro-donating mesomeric effect of a single aniline’s nitrogen through an amidation reaction. This target was achieved by breaking the molecular symmetry through the conversion of one of its aniline groups into a corresponding anilide of pyroglutamic acid (PyroGlu). The objective was to explore how the inclusion of a single amide functionality would influence the electronic properties of the π-conjugated moieties to which it is attached and its capacity to interact with radical species. In fact, the conversion of an aniline group into an anilide should alter the DAN capability of promoting either a radical formation or instead scavenging newly formed radicals. This conversion was successfully accomplished through a simple solventless thermal treatment of a 1:1 mixture of DAN and PyroGlu at 160 °C, followed by a simple purification process leading to the desired target, the mono-pyroglutanilide. The oxidant and antioxidant properties of this asymmetric derivative were characterized using two standard assays: indocyanine green (ICG) for the pro-oxidative and 2,2-diphenyl-1-picrylhydrazyl radical (DPPH•) for the antioxidative. The results were then compared with the symmetrical DAN. To the best of our knowledge, this is the first work that tries to understand how an asymmetric amidation of a disubstituted naphthalene could affect its pro-oxidant/antioxidant properties.

## 2. Results

To modify the symmetry-related properties of 1,5-diamino-napthalene, we needed to explore a synthetic pathway for derivatizing a single aniline moiety while minimizing the self-polymerization that often occurs in liquid media, which can often lead to macromolecular structures [8]. Since we have recently investigated the flexibility of l-glutamic acid (Glu) in the low-temperature copolymerization of high-melting amino acids [9,10], we found it worthwhile to apply this approach to create an anilide derivative of DAN. In fact, when treated with Glu at 160 °C for 4 h, Glu converts into pyroglutamic acid (PyroGlu), a cyclic amide that melts and acts as an effective dispersing agent, thus avoiding the use of other solvents.

Intrigued by the idea of thermally treating a mixture of Glu and DAN at low temperatures, we conducted a preliminary TG/DSC analysis of DAN, both in its pure form and in a mixture with Glu and PyroGlu, to determine the most suitable thermal conditions to achieve our target.

**Figure 1:**
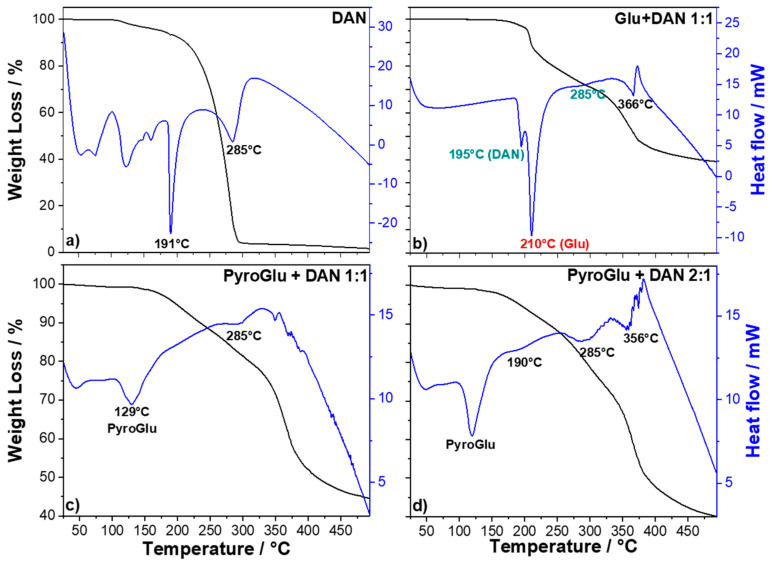
TG/DSC of pure 1,5-diamino-naphthalene (DAN) (**a**), 1:1 mixture of l-glutamic acid (Glu) and DAN (**b**), 1:1 mixture of pyroglutamic acid (PyroGlu) and DAN (**c**), 2:1 mixture of PyroGlu and DAN (**d**). Endothermic events downward.

The TG-DSC profile of DAN (Figure 1a) shows several endothermic events between 50 and 165 °C, associated with a 5% of weight loss, which can be assigned to the loss of adsorbed water and some phase transitions. 

**Scheme 2:**
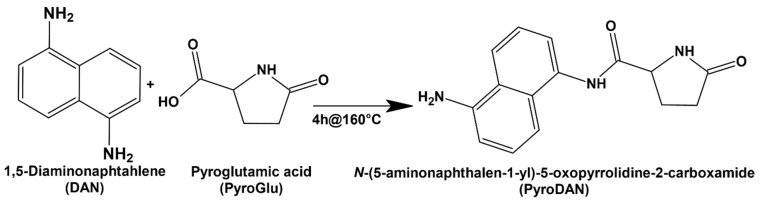
Synthetic scheme affording to PyroDAN.

**Figure 2:**
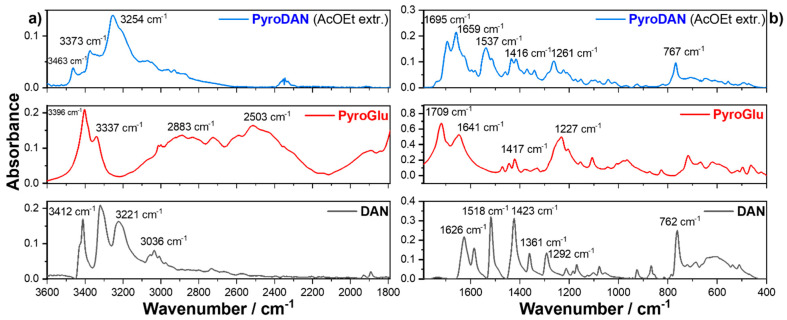
FTIR spectra of DAN (Sigma-Aldrich) (**bottom**), PyroGlu (Sigma-Aldrich) (**middle**), and PyroDAN (ethyl acetate extract) (**top**) in the range of 3600–1790 cm^−^^1^ (**a**) and 1790–400 cm^−^^1^ (**b**).

A sharp, intense endothermic event, peaking at 191 °C, marks the DAN melting point. After the melting process, the DAN molecules begin to degrade, as indicated by the subsequent endothermic event that peaks at 285 °C. Figure 1b shows the thermal behavior of a 1:1 mixture of DAN and Glu (the thermal degradation of Glu was thoroughly discussed in our previous work [9,10]). The main endothermic phenomena are the melting of DAN at 195 °C, associated with a negligible weight loss (≅4%), and a more intense peak at 210 °C, related to the melting of l-glutamic acid with a weight loss of about 12%. The latter event corresponds to a well-known dehydro-condensation process that leads to a cyclic amide of l-glutamic acid named pyroglutamic acid (PyroGlu) [10,11]. This transformation is highly dependent on the heating/time conditions and a complete conversion into PyroGlu can be obtained by treating l-glutamic acid at 160 °C for 4 h [12]. The other two slightly endothermic events, peaking at 285 and 366 °C, can be related to the degradation of the DAN and the glutamic derivatives, respectively. Since our synthetic strategy is based on reacting PyroGlu with DAN, we have decided to perform the reaction starting directly from commercially available PyroGlu to minimize the possible side reactions occurring between Glu and DAN before the conversion into PyroGlu. We have, therefore, analyzed the TG/DSC profile of two reaction mixtures obtained by combining PyroGlu and DAN in 1:1 (Figure 1c) and 2:1 (Figure 1d) ratios. The TG-DSC profiles of the mixtures show a different thermal response with respect to the mixture including Glu. A thermal event related to the melting of PyroGlu can be observed at around 130 °C in both mixtures with no weight loss associated. Interestingly, Figure 1c does not show any thermal event that can be correlated to DAN melting at 190 °C. This suggests that molten PyroGlu is acting as dispersing agent, thus promoting the reactivity of DAN, which shows a very little endothermic peak related to its thermal degradation (285 °C). The analysis of the 2:1 mixture (Figure 1d) shows again the event associated with the PyroGlu melting, a little shoulder at 190 °C for the DAN melting and two evident peaks associated with the thermal degradation of unreacted DAN (285 °C) and PyroGlu derivatives (366 °C). The apparent reduced reactivity of DAN in the 2:1 mixture could be possibly attributed to a more relevant self-reaction of PyroGlu since the weight loss of the two samples looks very similar. Based on the TG/DSC analysis, we have decided to pursue a synthetic pathway using a 1:1 ratio of DAN and PyroGlu, which acts as a dispersing agent, enabling the two compounds to react under mild thermal conditions (4 h at 160 °C) (Scheme 2).

After the reaction, the raw product was dispersed in milli-Q water, filtered, and the water dispersion was firstly purified by extracting the unreacted DAN with diethyl ether. Next, the residual water was then extracted with ethyl acetate, following the purification steps through TLC eluted with pure ethyl acetate. The final product was dried in an oven at 60 °C, resulting in a brownish-pink powder that was characterized using FTIR and NMR techniques. Figure 2 shows the FTIR absorption spectra of the ethyl acetate extract (top) compared with the spectra of pure PyroGlu (middle) and DAN (bottom).

The spectrum of DAN (Figure 2a bottom) is characterized by the asymmetric and symmetric stretching vibrations of the free aniline (-NH_2_), 3412 and 3319 cm^−1^, respectively, and by the asymmetric and symmetric stretching of aromatic C-H, 3220 and 3033 cm^−1^, respectively. The FT-IR spectrum of PyroGlu (Figure 2a middle) is also characterized by the presence of two N-H stretching vibrations, asymmetric (3396 cm^−1^) and symmetric (3333 cm^−1^), related to the pyrrolidine nitrogen of the lactam. The broad signal peaking around 2900 cm^−1^ can be related to O-H stretching of the free carboxylic acid whilst the broad signal peaking at 2500 cm^−1^ can be associated with the C-H stretching of the aliphatic moiety. The spectrum of the ethyl acetate extract is characterized by a couple of signals, 3463 and 3373 cm^−1^, which appear to be related, respectively, to the asymmetric and symmetric stretching of a free aniline group of a DAN moiety, though shifted to higher wavenumbers when compared with free DAN. The most prominent signal, peaking at 3254 cm^−1^, can be associated with the N-H stretching of an anilide group, thus indicating the formation of an amide bond between the carboxylic acid of PyroGlu and one of the anilines of the DAN. This assumption is further confirmed by the disappearance of the signal related to the free OH stretching of the carboxylic acid. The spectra in Figure 2b show the infrared profiles of the ethyl acetate extract in the region between 1790 and 400 cm^−1^. The FT-IR spectrum of DAN (Figure 2b bottom) is characterized by the presence of four peaks (1626, 1585, 1518, and 1423 cm^−1^) corresponding to the symmetric and asymmetric C_ar_=C_ar_ stretching of the aromatic backbone and N-H bending of the aniline moieties. The two signals at 1361 and 1292 cm^−1^ are instead related to the C-N symmetric and asymmetric stretching. Finally, the signal peaking at 762 cm^−1^ can be associated with the out-of-plane bending of three adjacent hydrogens [13]. The infrared spectrum of PyroGlu (Figure 2b middle) shows two infrared signals centered in 1709 and 1641 cm^−1^, which can be associated with the stretching of the free carboxyl (−C=O) moiety and the amide group of the γ-lactam (−CONH−), respectively. The tern of weak but sharp signals peaking at 1468, 1443, and 1417 cm^−1^ can be correlated with N-H, O-H, and C-H bending whilst the broad signal peaking at 1227 cm^−1^ can be related to the C-O stretching [14]. The analysis of the FTIR spectrum of the ethyl acetate extract (Figure 2 top), when compared with the infrared spectra of the precursor, highlights some structural insights that allow us to hypothesize an attribution to a mono-pyroglutamide derivative of the DAN, which we have named PyroDAN. In fact, the infrared spectrum of PyroDAN shows a clear decrease in the C=O stretching of the free carboxylate (1709 cm^−1^) in PyroGlu and the rise of C=O stretching in an Amide I bond (1643 cm^−1^), suggesting a reaction between an aniline of DAN and the carboxyl group of PyroGlu. This assumption is further confirmed by the presence of a new Amide II bond (1537 cm^−1^), the bending of PyroGlu (1416 cm^−1^), and the slightly shifted out-of-plane bending of the DAN (767 cm^−1^). Moreover, the C-O stretching of the PyroGlu free hydroxyl group (1227 cm^−1^) disappeared and the C-N stretching of the primary amine of DAN at 1292 cm^−1^ is replaced by the C-N stretching of a secondary C-N at 1261 cm^−1^ [13].

**Figure 3:**
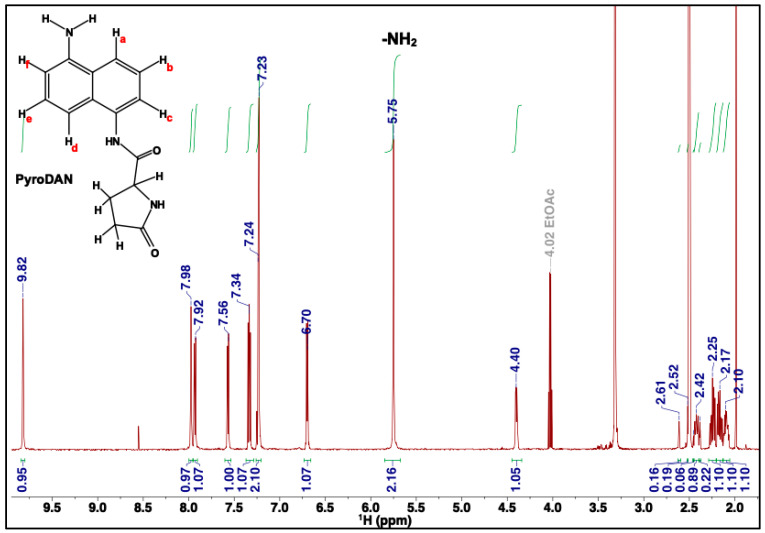
^1^H-NMR spectrum of Pyro-DAN in the range from 9.94 to 1.74 ppm.

**Figure 4:**
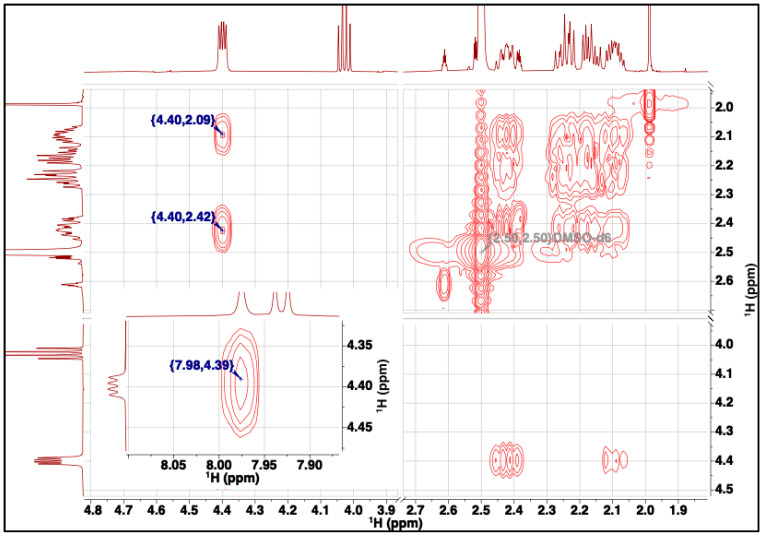
COSY-NMR of the pyroglutamic moiety of PyroDAN in the range of 1.8–4.8 ppm. The inset shows the region of 7.8–8.1 ppm. The NMR map was suitably cropped to improve its readability and overall organization.

The comparison of the FTIR absorption spectra presented in Figure 2 indicates that the ethyl acetate extract can reasonably be identified as a pyroglutamide derivative of DAN. To further clarify this identification, the ethyl acetate extract was structurally characterized using one-dimensional (1D) (Figure 3 and Figures S1 and S2) and two-dimensional (2D) (^1^H-^1^H COSY; ^1^H-^13^C HSQC; ^1^H-^15^N HSQC) (Figure 4 and Figures S5–S8) proton NMR spectroscopy in deuterated dimethyl sulfoxide (DMSO-*d*_6_). The 1D proton NMR spectra of the starting materials, 1,5-DAN and PyroGlu, were also reported as a reference in the Supplementary Information (Figures S3 and S4). Proton signals at 1.16, 1.99, and 4.02 ppm correspond to ethyl acetate residues resulting from the compound purification process [15].

The ^1^H-NMR shows the presence of two distinct moieties: the first, aromatic (see also Figure S1), related to DAN; and the second, aliphatic (see also Figure S2), related to pyroglutamic acid. The two moieties can be unambiguously assigned by taking advantage of the previous work performed by our group on pyroglutamic [9,10] and 1,5-diamino-naphthalene derivatives [8], as well as comparing the signals with those of the pure starting materials reported in the Supplementary Information (Figures S3 and S4).

The aminoacidic proton, α-CH, at 4.40 ppm, integrating for one ^1^H, confirms the presence of the pyroglutamic moiety. This signal is downshifted with respect to the same proton in unreacted Glu (3.87 ppm) because of its involvement in the formation of the internal amide, which is characteristic of the pyroglutamic structure. The presence of the lactam is established by the signal of the secondary amidic proton at 7.98 ppm, integrating for one ^1^H and, as evidenced from the COSY-NMR (inset Figure 4), clearly correlating with the α-CH proton. The formation of the five-membered ring is further proven by the presence of 2 γ-H at about 2.17 and 2.25 ppm, each integrating for one ^1^H.

Interestingly, the attribution of these two hydrogens is based upon their mutual coupling in the COSY-NMR, as well as their coupling with the two β-H around 2.10 and 2.40 ppm, which in turn are also clearly coupled with the α-CH (Figure 4). The latter are two hydrogens that become diastereotopic as a result of the lactam formation.

The analysis of the aromatic moiety of DAN highlights the presence of only a free aniline group evidenced by the signal at 5.75 ppm, integrating for two ^1^H. The second aniline group has, in fact, reacted with the free carbonyl of pyroglutamic acid to give an anilide, an aromatic amide, whose presence is confirmed by the signal at 9.82 ppm, integrating for one ^1^H. This is crucial to confirm that the thermal reaction of DAN with pyroglutamic acid at 160 °C afforded the formation of an asymmetric species (PyroDAN) instead of a symmetric trimer, such as that previously obtained reacting the two aniline groups of DAN with two molecules of l-glycine [8]. The reaction pattern affording to the PyroDAN molecule is reported in Scheme 2.

The aromatic region characteristic of diamino-naphthalene shows an asymmetric distribution of the benzenoid protons that suggests the presence of a partially reacted moiety. The signal at 6.70 ppm is attributed to the proton named **H_f_** (Figure 3), which couples with the nearby proton named **H_e_** (7.23 ppm). The signal at 7.23 ppm integrates for 2H because there is also a second proton with very similar chemical shift, 7.24 ppm. The analysis of the ^1^H-NMR clearly shows a tern of signals, 7.92, 7.56, and 7.34 ppm, correlated by an evident roofing effect. In particular, from the multiplicity, the signal at 7.34 ppm can be assigned to the proton named **H_b_**, thus the signal at 7.92 can be attributed to the proton closer to the anilide group, **H_c_**, and that at 7.56 ppm should be attributed to the proton **H_a_**. The only remaining signal, at 7.24 ppm, should be then related to the proton named **H_d_**, which is very close to the signal at 7.23 ppm, explaining why together they integrate for 2H. The HSQC analysis (Figures S5–S7), showing the one-bond heteronuclear correlation between ^1^H and ^13^C, also confirms the above attributions.

In particular, Figure S5 shows the correlation of the α-CH proton of the pyroglutamic moiety, at 4.40 ppm, with the carbon at 56.6 ppm. The correlation of the two γ-H of the PyroGlu moiety at about 2.17 and 2.25 ppm, with their corresponding ^13^C at 29.7 ppm and the ^1^H-^13^C correlation of the β-H with the carbon signal at 26 ppm, can be observed in Figure S6.

In the aromatic region, as reported in Figure S7, the assignment of the proton signal at 6.70 ppm to **H_f_** in Figure 1 is supported by the correlation with the carbon signal at 108.16 ppm. The two overlapping proton signals **H_e_** and **H_d_** can be more clearly resolved by the ^13^C signals of the attached carbons at 110.31 and 127.07 ppm, respectively. The HSQC cross-peak at 7.57/122.35 ppm in Figure S7 can be then assigned to the **H_a_** and the attached carbon, while the aromatic proton closer to the anilide group (**H_c_** in Figure 1) correlates with the carbon resonating at 120.41 ppm.

The mono-amidation of 1,5-DAN is further confirmed by a one-bond heteronuclear correlation (HSQC) between ^1^H and ^15^N (Figure S8), which shows the presence of three distinct nitrogen signals, 110.12, 121.53, and 123.41 ppm, correlated, respectively, to the free aniline (5.78 ppm), the lactam (7.99 ppm), and the anilide (9.83 ppm) of PyroDAN. An additional confirmation of the PyroDAN structure was obtained using HRMS-ESI+ spectra, which show a good matching between the calculated (270.12370) and experimental *m*/*z* (270.12354) for the molecular ion C_15_H_16_N_3_O_2_ [M+H]^+^ (Figure S9). A coherent fragmentation pattern of this molecular ion was obtained using higher-energy collisional dissociation (HCD) that highlights the breaking of the C-N and C-C bonds next to the hexocyclic carbonyl (Figure S10). This HCD MS-MS, following a loss of carbon dioxide, leads to two major fragments at *m*/*z* equal to 84 and 159, corresponding, respectively, to fragments from the pyroglutamic and DAN moieties.

**Figure 5:**
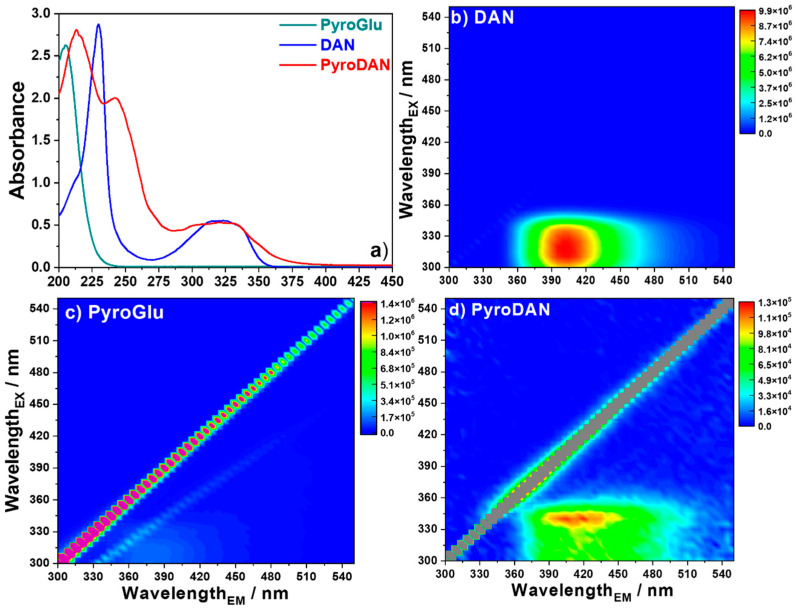
(**a**) UV-Vis of PyroGlu Sigma-Aldrich (dark cyan line), DAN (Sigma-Aldrich) (blue line), and PyroDAN (red line) in milli-Q water. 3D fluorescence map (x-emission; y-excitation; z-false color intensity scale) of DAN (Sigma-Aldrich) (**b**), PyroGlu (Sigma-Aldrich) (**c**), and PyroDAN (**d**) in milli-Q water.

After the NMR analysis, we compared the optical properties of PyroDAN to those of the starting materials (Figure 5).

Figure 5a shows the UV-Vis absorption spectra of PyroGlu, DAN, and PyroDAN. PyroGlu exhibits a strong absorption at around 202 nm (π→π* transition) due to the carbonyl moieties of the γ-lactam structure and the free carboxylic group. The absorption spectrum of DAN is characterized by a sharp strong band associated to a π→π* transition peaking at 230 nm with a small shoulder around 210 nm. A second broad band spanning between 275 and 360 nm and centered at 320 nm, is associated with the *n*→π* electronic transition, which results from the electron transfer from the lone pair of the nitrogen to the π* orbital of the naphthalene moiety. This broad band is formed by the combination of three bands peaking at 315, 325, and 334 nm (Figure S11). The poor resolution of the vibrational structure in the absorption spectrum can be correlated with the strong interaction between the amino group and a polar protic solvent, such as water. In fact, following Platt’s classification [16], in polar protic solvents these bands can be correlated with ^1^A_g_ → ^1^L_b_ transitions since, in presence of a hydrogen-bonding medium, the polar state ^1^L_a_ is more destabilized than ^1^L_b_ [17].

The absorption profile of PyroDAN is characterized by two strong bands peaking first at 213 nm and ascribed to the π→π* electronic transitions due to the carbonyl moieties of the γ-lactam structure and the new anilide moiety. A second absorption band, inherited from the DAN aromatic moiety, is slightly red-shifted at 242 nm with respect to the parent structure due to the introduction of a new amide bond. The characteristic combined broad band associated with the *n*→π* electronic transition of the DAN, spanning from 290 to 340 nm, became wider and more structured when compared with DAN (Figure S11). This broadening effect has been already observed for pyrenyl-amides by Vullev and colleagues, who discovered that amides, though breaking the pyrene symmetry, cause a small shift in both UV-Vis absorption and the fluorescence spectra [5]. In fact, the peaks at 315 and 325 nm appeared both blue-shifted in PyroDAN, respectively, to 302 and 320 nm. In addition, two new signals, a shoulder peaking at 275 and a little band peaking 289 nm, can be associated most probably with the *n*→π* electronic transition of the two carbonyl groups, the intra- and inter-molecular amides of the PyroGlu moiety. It is noteworthy that the changes in the fine structure of the long-wavelength band are ascribable to the alteration of the DAN symmetry due to the transformation of a single aniline to anilide operated by the PyroGlu moiety. In fact, the total dipole length of DAN is characterized by the bond moment of the individual amino groups that act in opposite directions to each other [17]. The transformation of a single aniline into anilide breaks this symmetry, thus increasing the molecular dipole by replacing one of the anilines with a functional group, the pyroglutamide, characterized by a reduced electro-donating mesomeric effect when compared with the unreacted amino group.

**Figure 6:**
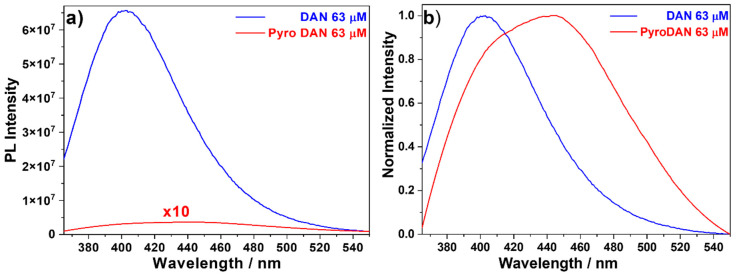
Fluorescence spectra of two 63 µM solutions of DAN (blue curve) and PyroDAN (red curve), in milli-Q water, acquired in the range of 365–550 nm at the maximum excitation wavelength (λ_ex_ of 320 nm) (**a**). The spectrum of PyroDAN was multiplied by a factor of 10 for an easier comparison with DAN; fluorescence spectra of curve (**a**) normalised to 1 (**b**).

The 3D fluorescence map (x-emission; y-excitation; z-false color intensity scale) of DAN (Figure 5b) is characterized by a strong emission centered at 405 nm (λ_ex_ = 320 nm), in accordance with the previous findings [17]. PyroDAN exhibits a broad fluorescent emission centered around 415 nm with an excitation maximum at 340 nm (Figure 5d). In contrast, commercial PyroGlu does not display any significant emission (Figure 5c). The fluorescence data suggest that the photoluminescence observed in PyroDAN is related to the conjugated bonds present in the aromatic moiety inherited from the DAN. However, the original fluorescence intensity of the DAN resulted in a dramatic reduction due to the electronic effect of the pyroglutamic anilide on the π-conjugation. Moreover, the small spectral broadening evidenced in the PyroDAN fluorescence confirms what was already found for the fluorescence spectra of pyrenyl-amides [5]. To better understand how the structural modification introduced in PyroDAN affected its quantitative fluorescence emission in comparison with DAN, we have also acquired a fluorescence spectrum for DAN and PyroDAN at the same molar concentration equal to 63 µM in milli-Q water (Figure 6), so as to compare directly the emissions in a more quantitative manner.

Figure 6a shows clearly that, at the same concentration, the intensity of DAN is about 200 times higher than PyroDAN. Given the high difference in intensity, the spectrum of PyroDAN needed to be multiplied by a factor of 10 to avoid being confused with the zero line. This phenomenon can be seen as a direct consequence of the electronic perturbation of the π-conjugation of the DAN backbone due to the functional transformation of a single aniline into a pyroglutamyl anilide, which decreases the electro-donating effect of its nitrogen lone pair. Moreover, after normalizing the two spectra of Figure 6a to 1 (Figure 6b), the differences in the fluorescence profile of the two molecular structures appear with more clarity. In fact, while the curve profile of DAN can be seen as a result of a single component with a maximum centered a 403 nm, the profile of PyroDAN is clearly resulting from a combination of at least two components: one closely related to the DAN moiety and a second centered at 445 nm. While the first component can be ascribed to a radiative decay internal to the π-conjugated system, the second band can be due to the presence of an additional moiety external to the π-conjugated system.

The fine structure of PyroDAN shown in Figure 6b confirms what was already discussed above for the absorption profile of PyroDAN; in particular, the signal broadening of the long-wavelength band due to a desymmetrization of the original DAN with a charge transfer extended from the π-conjugated naphthalene backbone to the pyroglutamyl moiety.

### 2.1. Oxidant Activity

**Scheme 3:**
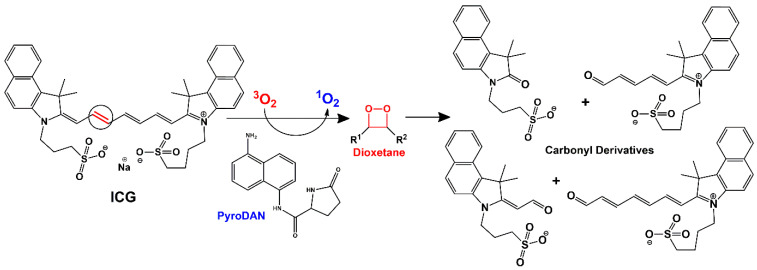
ICG photodegradation reaction induced by singlet-oxygen-generated irradiating PyroDAN at 320 nm. Adapted from reference [18].

After analyzing the differences in the photoluminescence properties of PyroDAN in relationship with the parent structure of DAN, we have deemed it of interest to understand how the symmetry breaking due to the amidation could affect its activity. In a recent work, we have investigated the pro-oxidant activity of carbon dots obtained by reacting DAN with l-glycine. This activity is promoted by the generation of singlet oxygen, ^1^O_2_, a strong oxidant reactive oxygen species (ROS), and it can be monitored using the only near infra-red (NIR) probe approved by the Food and Drug Administration (FDA) of the USA, which is the indocyanine green (ICG) [18]. ICG is a water-soluble tricarbocyanine dye (Scheme 3) that displays a maximum absorption peak centered at 780 nm (Figure S12). The irradiation of the photosensitizer generates a singlet oxygen, which induces the molecular degradation of the ICG conjugated polyolefin structure via dioxetane formation with subsequent oxidative fragmentation into several carbonyl derivatives [18]. The fragmentation can be monitored by following the decrease in ICG absorption during light irradiation, thus monitoring the singlet oxygen effect produced by a specific photosensitizer.

Indeed, DAN is responsible for energy transfer (type II reaction) towards molecular oxygen in its triplet ground state, ^3^O_2._ This process is followed by the spin pairing of the electrons in the π* antibonding orbitals with the generation of highly reactive singlet oxygen, with irrelevant contributions of other ROS [8,18,19].

**Figure 7:**
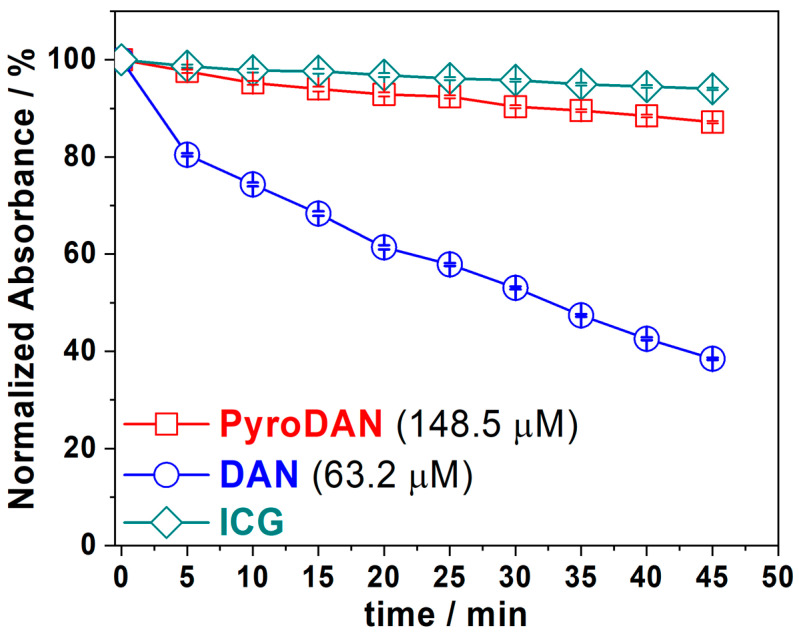
ICG singlet oxygen assay for PyroDAN (red line), DAN (blue line), and reference ICG (dark cyan). The error bars are shown inside the symbols. The lines are a guide for the eyes.

The oxidant activity of PyroDAN was also studied using the ICG photodegradation protocol and the results were compared with those of DAN (Figure 7). This assay was performed by irradiating the photosensitizers (DAN or PyroDAN) with a fixed wavelength of 320 nm. The oxidant activity of the photosensitizer is evaluated as percentage of the ICG decomposition against time, measuring the absorption of residual ICG at 780 nm. In order to evaluate the background decomposition of ICG, a control solution of ICG was monitored at the same time intervals as the samples, and the error associated was estimated as a standard deviation of a triplicate set of measurements. Figure 7 shows the ICG degradation curves for the ICG, ICG + DAN, and ICG + Pyro DAN, as a function of time.

The graph shows that derivatizing DAN as a mono-pyroglutanilide affects its capability of generating singlet oxygen and thus its oxidant activity. The PyroDAN profile closely resembles that of bare ICG, which serves as a control and shows only a 4% degradation of ICG within 45 min. In contrast, DAN is capable of oxidizing nearly 60% of ICG in the same time frame. Additionally, achieving this lower level of activity requires a concentration of PyroDAN that is over twice that of DAN. In conclusion, the derivatization of DAN into mono-pyroglutanilide almost completely suppresses its oxidizing capabilities. This likely occurs due to the symmetry breaking, with consequent electron density perturbation, resulting from the amidation of only one of the aniline moieties, which hampers the possibility of generating singlet oxygen.

### 2.2. Antioxidant Activity

**Scheme 4:**
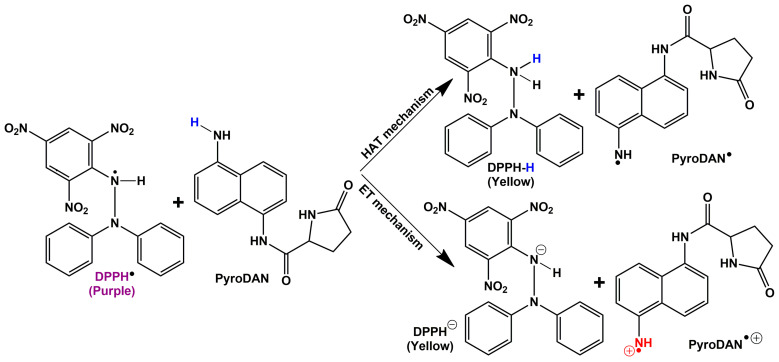
DPPH• radical scavenging for PyroDAN. Adapted from reference [4].

The antioxidant activity of PyroDAN was assessed using a standard DPPH• assay to determine the scavenging activity at various concentrations and compare the result with that of pure DAN. The assay is based on the capability of the sample to quench the stable radical DPPH• (2,2-diphenyl-1-picrylhydrazyl) by releasing either an atom of hydrogen (H•) through a mechanism known as hydrogen atom transfer (HAT) or through an electron transfer (ET). The latter can be of two kinds: single electron transfer followed by proton transfer (SET-PT) or sequential proton loss electron transfer (SPLET) [4]. The assay is performed by monitoring the decrease of the UV-Vis absorption of the DPPH at 524 nm with a corresponding color shift from purple to pale yellow (Figures S13 and S14). Scheme 4 illustrates these mechanisms applied to PyroDAN.

**Figure 8:**
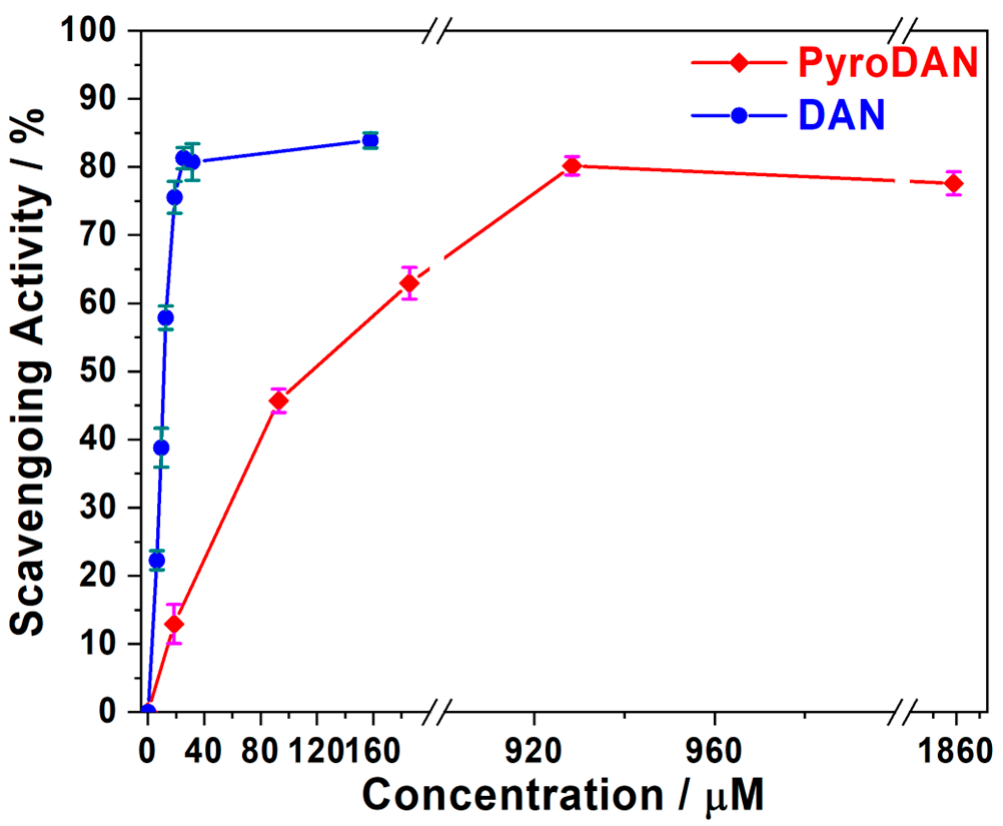
DPPH• scavenging activity of DAN (blue curve) with associated error bars (dark cyan) and PyroDAN (red curve) with associated error bars (dark magenta). The lines are a guide for the eyes.

Figure 8 illustrates the antioxidant activity of DAN and PyroDAN resulting from the DPPH• assay.

The percentage of scavenging activity is shown as a function of the increasing concentrations of antioxidants (DAN or PyroDAN). At concentrations as low as 20 µM, DAN exhibits a significantly higher antioxidant activity, neutralizing about 80% of the DPPH• radical. In contrast, PyroDAN only achieves this level of neutralization at a much higher concentration of 930 µM, indicating that DAN is approximately 45 times more efficient than its mono-pyroglutanilide counterpart. Interestingly, although the PyroDAN antioxidant activity is considerably weaker, some activity is still retained. The transformation of aniline into anilide, with the subsequent alteration of the electronic environment of the aromatic structures, greatly reduces the PyroDAN capability of hydrogen donation. An explanation can be found in the change of resonance structures characterizing the asymmetric PyroDAN with respect to the highly symmetric and planar DAN. The lone pair of the amidic nitrogen, in fact, can be delocalized either on the aromatic ring, increasing its electron density, or on the amidic carbonyl, generating a positively charged nitrogen that could drain out electron density from the π-conjugate system.

**Scheme 5:**
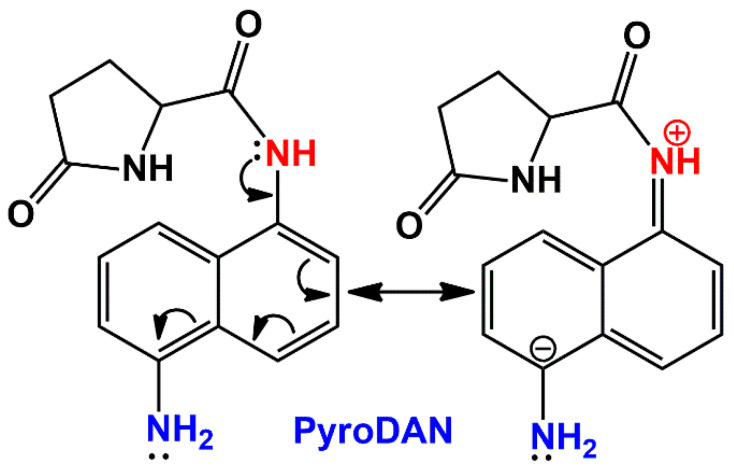
Some resonance structures of PyroDAN.

The mesomeric electro-donating effect (Scheme 5) is predominant and thus the increased electron density on the π-conjugated system, paired with the symmetry breaking, makes the aniline nitrogen more basic and the hydrogen less acidic and less prone to homolitically dissociate and join the free DPPH• radical. Moreover, although the previous literature reported the bland antioxidant activity of some acetanilides [19], in our case, the already poor acidic hydrogen of the anilide group (pK_a_ ≅ 18) is less disposed to dissociate homolitically.

### 2.3. Conclusions

This study demonstrates the successful synthesis and characterization of a novel mono-pyroglutanilide derivative of 1,5-diamino-naphthalene (DAN), PyroDAN, via a solventless thermal coupling with l-pyroglutamic acid. The derivatization process effectively modified the electronic and reactive properties of DAN, as evidenced by the extensive spectroscopic and analytical evaluations.

The antioxidant properties, while significantly reduced when compared with DAN, are still retained at a mild level, as shown by the DPPH• assay. Importantly, the oxidant activity of PyroDAN was almost entirely suppressed, as confirmed by the ICG photodegradation experiment. These results indicate that the symmetry-breaking modification of the DAN structure has a profound impact on its redox properties.

To the best of our knowledge, this is the first work that tries to understand how an asymmetric amidation of a diamino-naphthalene could affect its pro-oxidant/antioxidant properties. The tuneable fluorescent properties of PyroDAN—the mild antioxidant activity and the inhibition of the cytologically harmful pro-oxidant properties—suggest promising applications in bioimaging and other biological fields. Future research will focus on further exploring its biological compatibility and performance in targeted applications.

## 3. Materials and Methods

### 3.1. Chemicals

l-glutamic acid (2-aminopentanedioic acid) powder (Sigma-Aldrich, ≥99%), l-pyroglutamic acid ((*S*)-(−)-2-pyrrolidone-5-carboxylic acid) powder (Sigma-Aldrich, ≥99%, C_5_H_7_NO_3_), and 1,5-diamino-naphthalene (1,5-naphthalenediamine) powder (Sigma-Aldrich, ≥97%, C_10_H_6_(NH_2_)_2_) were purchased from Sigma Aldrich, St Louis, MO, USA. Chemicals were used without further purification. Milli-Q water was used for the synthesis and analysis. Diethyl ether was purchased from Merck KGaA, Darmastadt, Germany. Ethyl acetate was purchased from Carlo Erba reagents (Cornaredo, Italy).

### 3.2. Materials Synthesis

#### Synthesis of *N*-(5-aminonaphthalen-1-yl)-5-oxopyrrolidine-2-carboxamide (PyroDAN)

l-pyroglutamic acid (0.5 g) and 1,5-diamino-naphthalene (0.613 g, 1 eq.) were mixed in a mortar and then transferred into a crucible that was placed in oven with a 10 °C/min ramp to reach a temperature of 160 °C, which was held for 4 h. The black glassy solid was allowed to cool down to room temperature, dispersed in 20 mL of milli-Q water and sonicated for 15 min. The water dispersion was filtrated using a 0.45 µm syringe filter (Whatman Puradisc 30/0.45, Sigma Aldrich, Europe) and the resulting homogeneous water solution was collected and transferred into a 250 mL separatory funnel. The residual unreacted DAN was completely extracted from the water dispersion using a 30 mL aliquot of diethyl ether for each extraction until the complete removal of DAN (at least 4 × 30 mL). The presence of DAN was monitored using TLC eluting with pure ethyl acetate. The water residue was then extracted with ethyl acetate to collect the desired product (PyroDAN) using 30 mL aliquot of ethyl acetate for each extraction (at least 4 × 30 mL) and then monitoring using TLC eluting with pure ethyl acetate. The fractions of ethyl acetate were then collected and evaporated using a rotary evaporator to produce a brownish pink powder, whose yield, calculated with respect to the starting materials, was 10% *w*/*w*. The ethyl acetate extract was characterized using FT-IR, NMR (DMSO-*d*_6_), UV-Vis, and fluorescence spectroscopies.

### 3.3. Materials Characterization

***Thermal analysis***. A combined thermogravimetric (TG)—differential scanning calorimetry (DSC) analysis was performed using an SDT Q600 (TA instruments, Milano, Italy). All the analyses were performed under N_2_ inert atmosphere (flow rate 20 mL min^−1^) with 10 °C min^−1^ heating rate up to 500 °C.

***Infrared analysis.*** A Fourier-transform infrared (FTIR) analysis was performed using an infrared Vertex 70 interferometer (Bruker, Milano, Italy). The FTIR absorption spectra were recorded in the 4000–400 cm^−1^ range with a 4 cm^−1^ resolution and 128 scans. The spectra were acquired using potassium bromide pellets (KBr, ≥99.5%, Fluka, Milano, Italy).

***UV-Vis analysis***. The UV-Vis absorption spectra were recorded using a Nicolet Evolution 300 UV-Vis spectrophotometer (Thermo Fisher, Milano, Italy) with a bandwidth of 1.5 nm in the range from 200 to 900 nm.

***Photoluminescence.*** The spectroscopy measurements were performed on a Horiba Jobin Yvon NanoLog (Kyoto, Tokyo). 3D photoluminescence (PL) maps of the aqueous solutions were recorded from 250 nm to 600 nm (slit 1 nm, integration time 0.2 s). The same spectrofluorometer and identical parameters settings were used in all the analyses.

***Nuclear Magnetic Resonance.*** The NMR spectra were collected at 298 K using a Bruker (Bruker Biospin, Karlsruhe, Germany) Avance spectrometer, equipped with a 5-mm multinuclear inverse detection probe BBI, in deuterated dimethyl sulfoxide (99.8%, Cambridge Isotope Laboratories Inc., Andover, MA, USA) at frequencies of 600.13 and 150.90 MHz for ^1^H and ^13^C NMR, respectively. One-dimensional (1D) proton spectra were acquired with a pulse of 10.00 µs and 64 scans. A gradient-enhanced magnitude-mode ^1^H-^1^H COSY map was obtained by acquiring 2048 points FID and 256 increments, 32 scans over 6600 Hz spectral windows in both dimensions. Heterocorrelated ^1^H-^13^C HSQC and ^1^H-^15^N HSQC were acquired with similar parameters, taking a 220 ppm carbon spectral window and 140 ppm nitrogen spectral window, respectively.

***High Resolution Mass Spectrometry Electrospray Ionization.*** The HRMS-ESI spectra were acquired using a Thermo Finnigan Q Exactive spectrometer (Thermo Fisher Scientific, Waltham, MA, USA) with an API-HESI source and a Fourier transform orbital trap (Orbitrap, Thermo Finnigan, Waltham, MA, USA). The samples were introduced as acetonitrile solutions at a 0.1 mg/L concentration.

***Evaluation of the radical scavenging activity trough DPPH***^●^***assay.*** The radical DPPH• (2,2-diphenyl-1-picrylhydrazyl) assay was performed following a previously published procedure with some modifications [20]. A stock solution of DPPH• (250 µM in methanol) was prepared and stored at 4 °C in the dark. A 50 µg mL^−1^ stock solution (316 µM) of DAN was used to prepare several dilutions of DAN in milli-Q water. Later, mixtures composed of 250 μL of DPPH• methanol solution (125 µM final concentration) and 250 μL of DAN water solution were prepared for each DAN dilution with final concentrations of 6.3, 9.5, 12.6, 18.9, 25.3, 31.6, and 158 μM. The mixtures were stored in the dark at room temperature for one hour prior to analysis. After the incubation time, the UV-visible spectra of the methanol/water solutions were acquired in a volume of 300 μL into quartz test cuvette with a light path of 1 mm using the Nicolet Evolution 300 spectrophotometer in the range from 200 to 900 nm and with a bandwidth of 1.5 nm. A blank sample was prepared using a methanol/water mixture in a 1:1 ratio and an analog mixture was used as the control of the experiment by adding a 125 µM final concentration of DPPH•. Therefore, the following formula was applied to calculate the radical scavenging activity (RSA) of DAN in the different tested concentrations:

(1) $$\mathrm{RSA}\;(\%)\;=\;\left(\frac{{\mathrm{CA}}_{524}-\;{\mathrm{SA}}_{524}}{{\mathrm{CA}}_{524}}\right)\;\times \;100$$

with CA and SA as the absorbance at 524 nm for the control (C) and for the samples (S). Finally, the DPPH• scavenging activity was evaluated by plotting the percentage of radical inhibition against DAN concentration. The same procedure was adopted to evaluate the antioxidant activity of PyroDAN with concentrations ranging from 0.01 to 0.1 mg mL^−1^. The error bars were estimated as a standard deviation of a triplicate set of measurements.

### 3.4. Evaluation of the Singlet Oxygen ^1^O_2_ Production by Indocyanine Green Assay

The light-driven singlet oxygen (^1^O_2_) evolution from DAN and PyroDAN was assessed through the photodegradation of indocyanine green (ICG). A water solution of DAN (63.2 µM) or PyroDAN (148.5 µM) was mixed with a water solution of ICG (6.45 µM) using milli-Q water as a solvent and transferred in a 3 mL quartz-test cuvette. The assay was performed using a NanoLog Horiba Jobin Yvon spectrofluorometer equipped with a 450 W Xenon lamp for the irradiation of the mixture (λ_ex_ = 320 nm to match with DAN absorption) in real-time control mode with a 2 nm slit aperture. The UV-Vis spectra of the mixture were recorded at t_0_ and every 5 min of irradiation up to 45 min using the Nicolet Evolution 300 UV-Vis spectrophotometer with a bandwidth of 1.5 nm from 200 to 900 nm. The photoinduced degradation of ICG was monitored plotting the values of normalized absorbance at 780 nm (%) against time (min). The photostability of ICG alone was performed with the same procedure as the control of the experiment. The error bars were estimated as a standard deviation of a triplicate set of measurements.

## Data Availability

The original contributions presented in this study are included in the article/Supplementary Materials. Further inquiries can be directed to the corresponding authors.

## Supplementary Materials

The following supporting information can be downloaded at: https://www.mdpi.com/article/10.3390/molecules30081802/s1, Figure S1. 1D ^1^H NMR spectrum of PyroDAN in the range from 9.95 to 4.30 ppm; Figure S2. 1D ^1^H NMR spectrum of PyroDAN in the range from 2.65 to 2.03 ppm; Figure S3. 1D ^1^H NMR spectrum of 1,5-DAN (DMSO-*d*_6_) in the range from 9.70 to 1.5 ppm. Inset with the expansion of the region of 7.3–6.5 ppm; Figure S4. 1D ^1^H NMR spectrum of PyroGlu (DMSO-*d*_6_) in the range from 8.1 to 1.8 ppm. Inset of the region of 1.85–2.40 ppm; Figure S5. ^1^H-^13^C HSQC spectrum of PyroDAN (DMSO-*d*_6_) in the range of 0.3–9.5 ppm (^1^H) and 0–140 ppm (^13^C); Figure S6. ^1^H-^13^C HSQC spectrum of PyroDAN (DMSO-*d*_6_) in the range of 2.77–1.75 ppm (^1^H) and 24–33 ppm (^13^C); Figure S7. ^1^H-^13^C HSQC spectrum of PyroDAN (DMSO-*d*_6_) in the range of 8.3–6.5 ppm (^1^H) and 107–129 ppm (^13^C); Figure S8. ^1^H-*^15^*N HSQC spectrum of PyroDAN (DMSO-*d*_6_) in the range of 10.3–5.5 ppm (^1^H) and 101–125 ppm (^15^N); Figure S9. HRMS (ESI+) *m*/*z* calculated (bottom) for C_15_H_16_N_3_O_2_ [M+H]^+^: 270.12370, found for PyroDAN (top): 270.12354 (Δ = −0.6 ppm). Figure S10. Higher-energy collisional dissociation (HCD) HiRes MS-MS (ESI+) spectrum of PyroDAN; Figure S11. Expansion of the UV-Vis of DAN (blue line) and PyroDAN (red line) in water for the range of 286–345 nm; Figure S12. UV-Vis absorption spectra of ICG+DAN (a) and ICG + PyroDAN (b) as a function of time; Figure S13. UV-Vis absorption spectra of DPPH with several concentrations of PyroDAN ranging from 0.01 to 0.1 mg/mL; Figure S14. UV-Vis absorption spectra of DPPH with several concentrations of DAN ranging from 6.3 to 158 µM.
