# Supplementary material for: Tuning the Activity of 1,5-Diamino-naphthalene Through an Asymmetric Mono-Amidation with Pyroglutamic Acid
Source: Molecules. 2025 Apr 17;30(8):1802. doi: 10.3390/molecules30081802 (PMC12029918; doi:10.3390/molecules30081802)
Supplement: Supplementary file 1 [file molecules-30-01802-s001.zip › molecules-3481871-supplementary.pdf]

Tuning the Activity of 1,5-Diamino-Naphthalene through an Asymmetric Mono-Amidation with Pyroglutamic Acid

Davide Carboni,[*1] Marta Cadeddu,[1] Federico Olia,[1] Federico Fiori,[1], Roberto Anedda,[2] Massimo Carraro,[3] Luca Malfatti[1] and Plinio Innocenzi[*1]

[1]Laboratory of Materials Science and Nanotechnology, CR-INSTM, Department of Biomedical Sciences, University of Sassari, Viale San Pietro 43/B, Sassari 07100. Italy.

[2]Porto Conte Ricerche. Strada Provinciale 55, Porto Conte Capo Caccia, km. 8,400. 07041 Alghero, Italy

[3]Dipartimento di Scienze Chimiche, Fisiche, Matematiche e Naturali, Università degli Studi di Sassari, via Vienna 2, 07100 Sassari, Italy

* Corresponding Authors: dcarboni@uniss.it and plinio@uniss.it

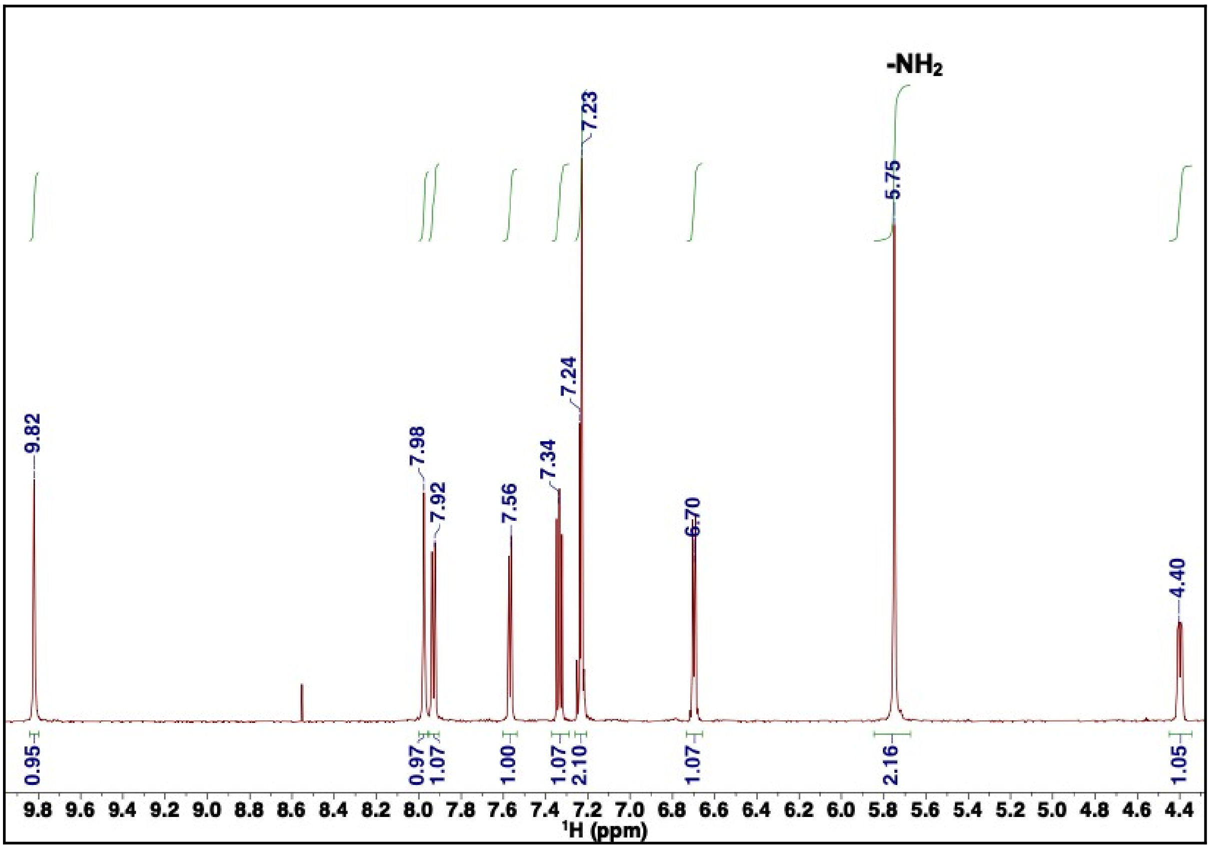

Figure S1. 1D ^{1}H NMR spectrum of PyroDAN in the range 9.95 to 4.30 ppm.

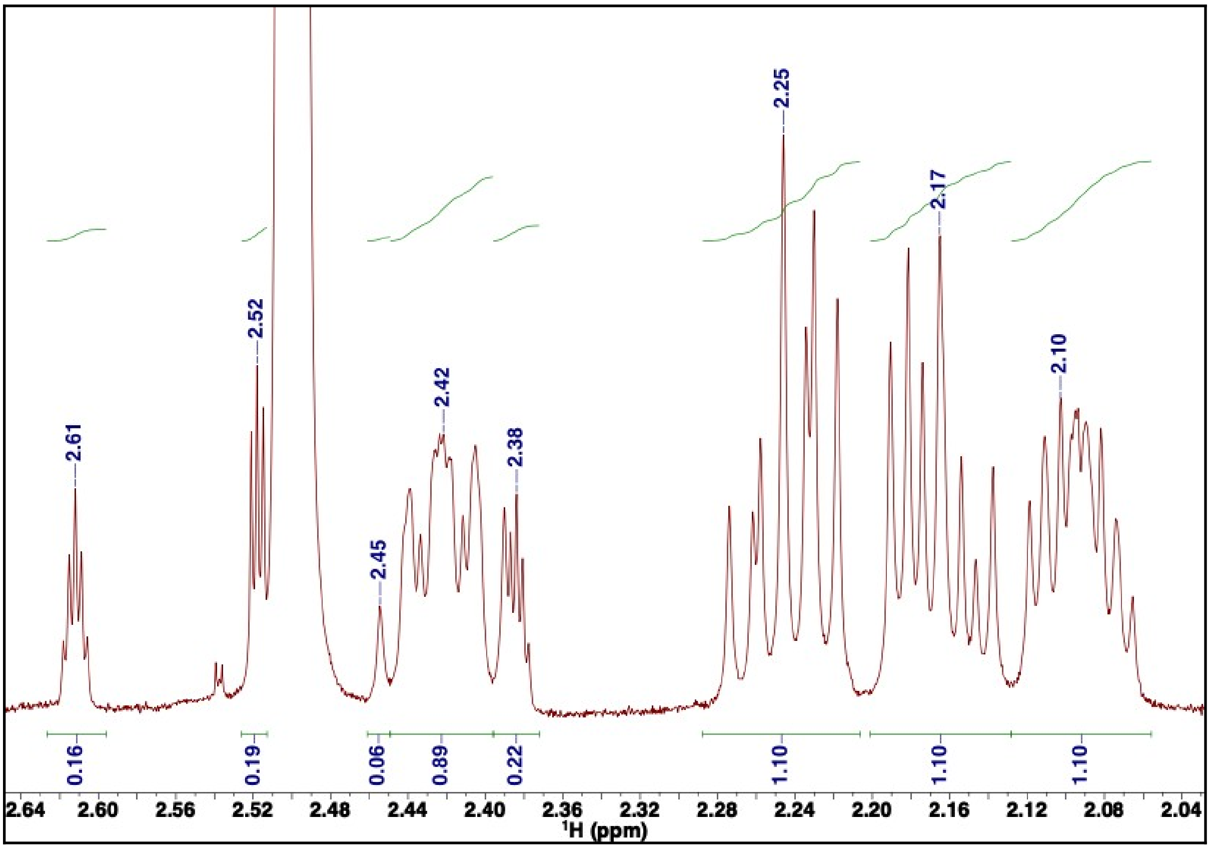

Figure S2. 1D ^{1}H NMR spectrum of PyroDAN in the range 2.65 to 2.03 ppm.

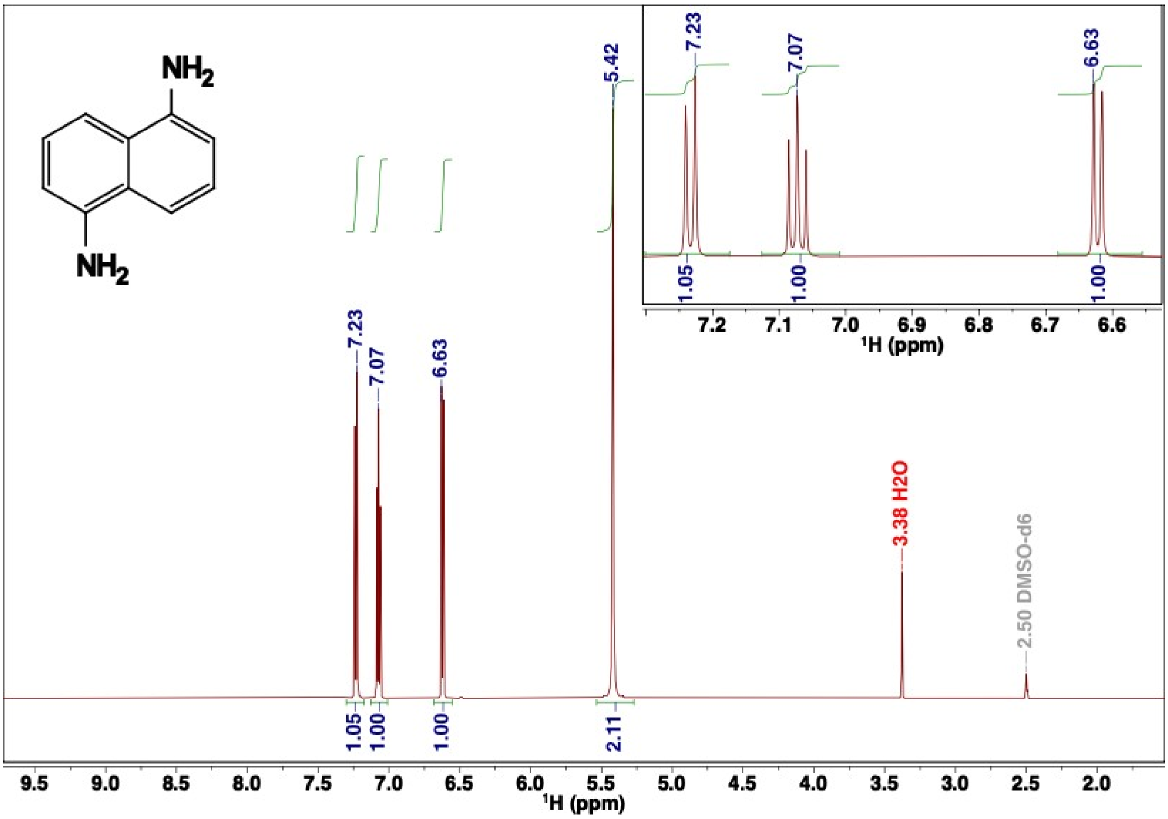

Figure S3. 1D ^{1}H NMR spectrum of 1,5-DAN (DMSO-d6) in the range from 9.70 to 1.5 ppm. Inset with the expansion of region 7.3-6.5 ppm.

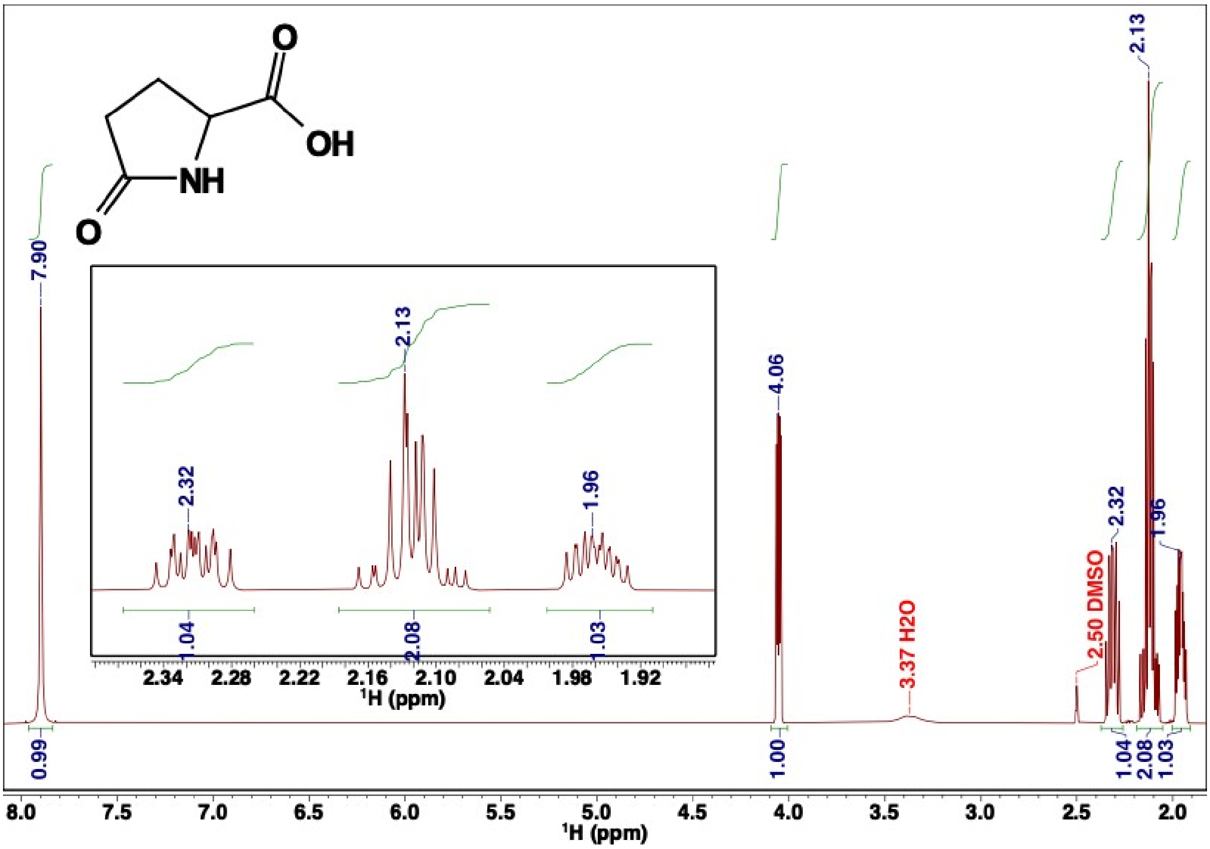

Figure S4. 1D ^{1}H NMR spectrum of PyroGlu (DMSO-d6) in the range from 8.1 to 1.8 ppm. Inset of region 1.85-2.40 ppm.

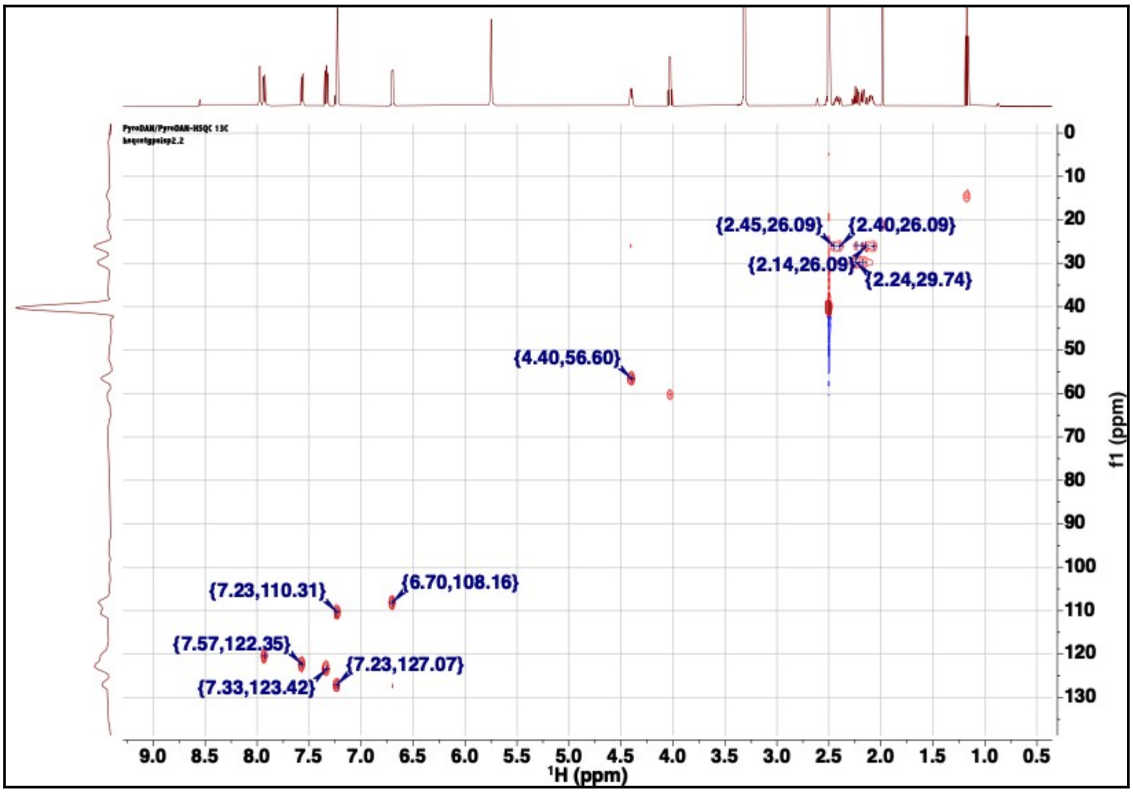

Figure S5. ^{1}H-^{13}C HSQC spectrum of PyroDAN (DMSO-d6) in the range 0.3-9.5 ppm (^{1}H) and 0-140 ppm (^{13}C).

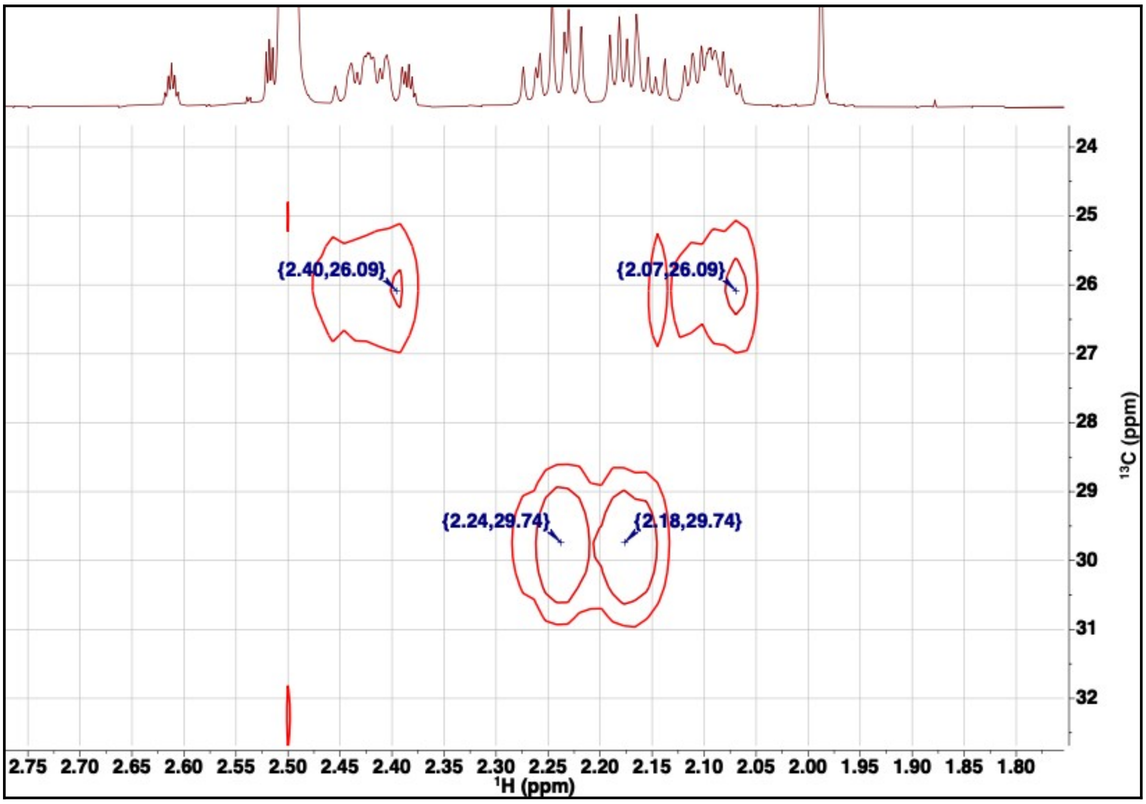

Figure S6. ^{1}H-^{13}C HSQC spectrum of PyroDAN (DMSO-d6) in the range 2.77-1.75 ppm (^{1}H) and 24-33 ppm (^{13}C).

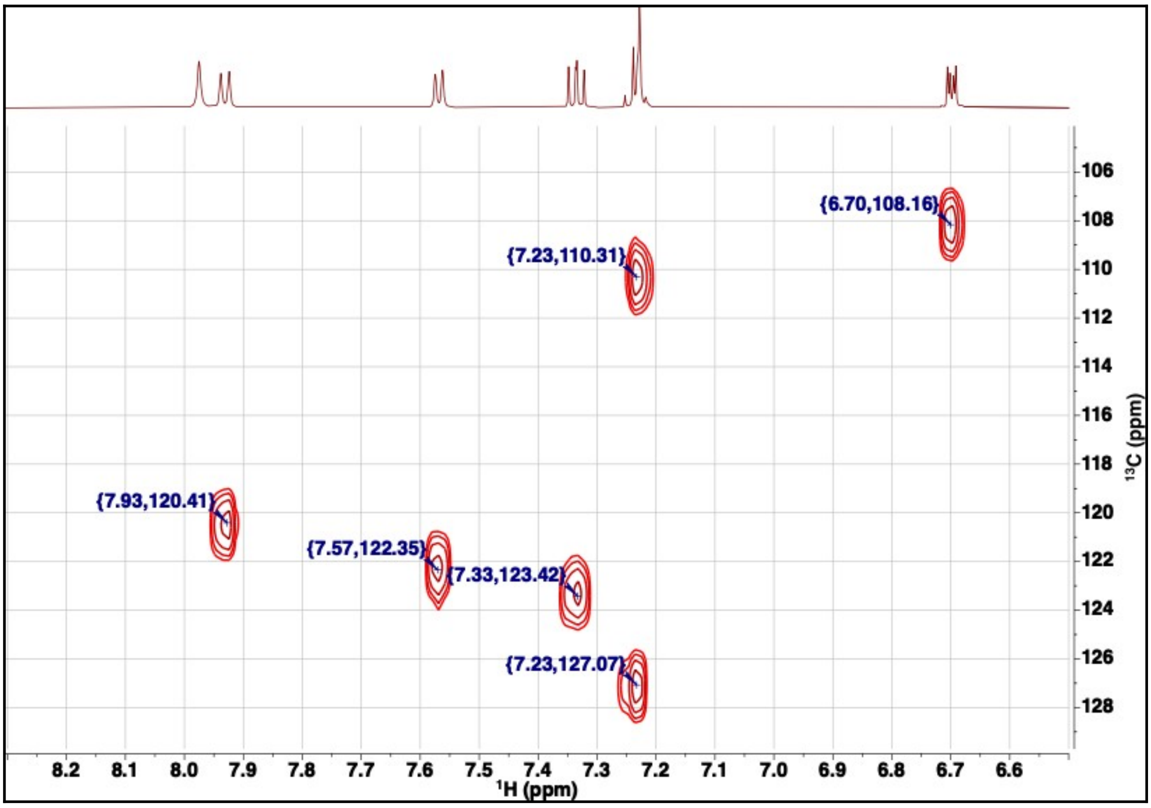

Figure S7. ^{1}H-^{13}C HSQC spectrum of PyroDAN (DMSO-d6) in the range 8.3-6.5 ppm (^{1}H) and 107-129 ppm (^{13}C).

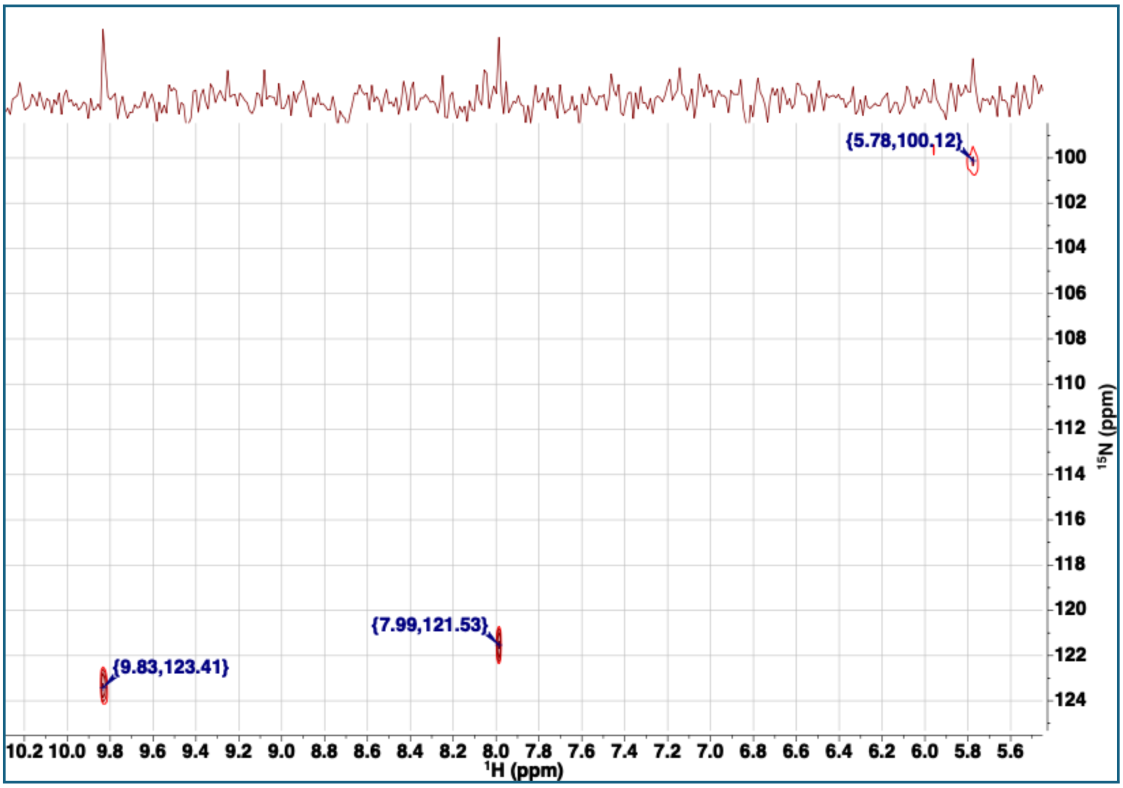

Figure S8. ^{1}H-^{15}N HSQC spectrum of PyroDAN (DMSO-d6) in the range 10.3-5.5 ppm (^{1}H) and 101-125 ppm (^{15}N).

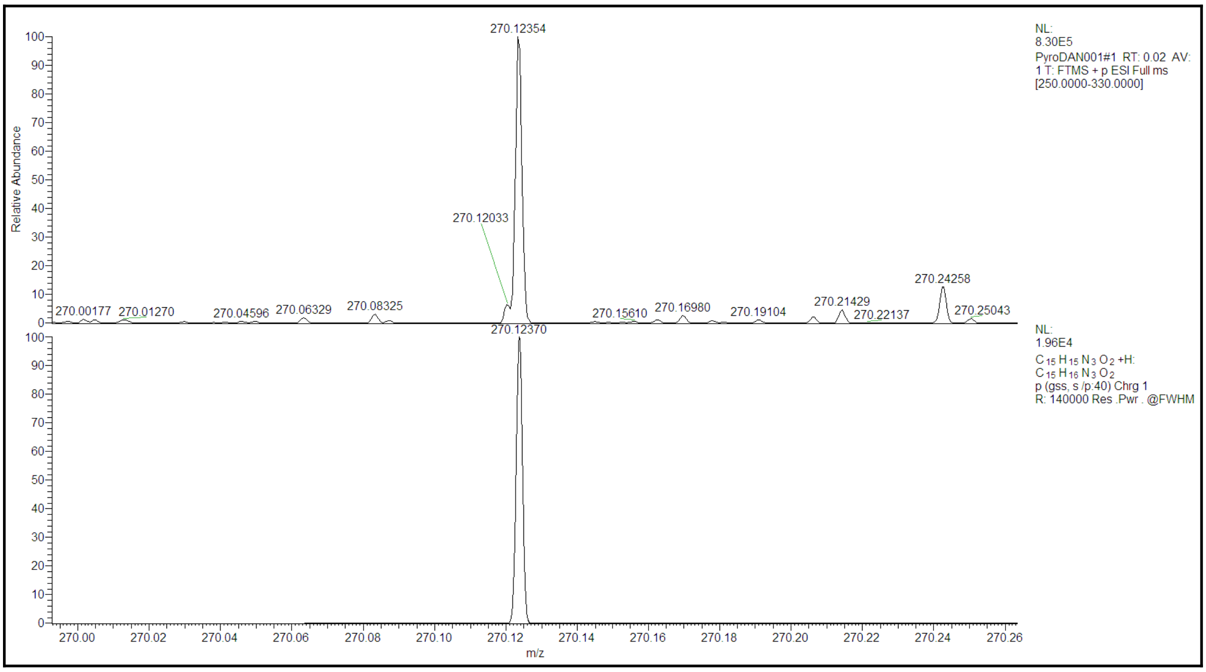

Figure S9. HRMS (ESI+) *m*/*z* calculated (**bottom**) for $C_{15}H_{16}N_3O_2$ $[M+H]^+$: 270.12370, found for PyroDAN (**top**): 270.12354 (Δ = −0.6 ppm).

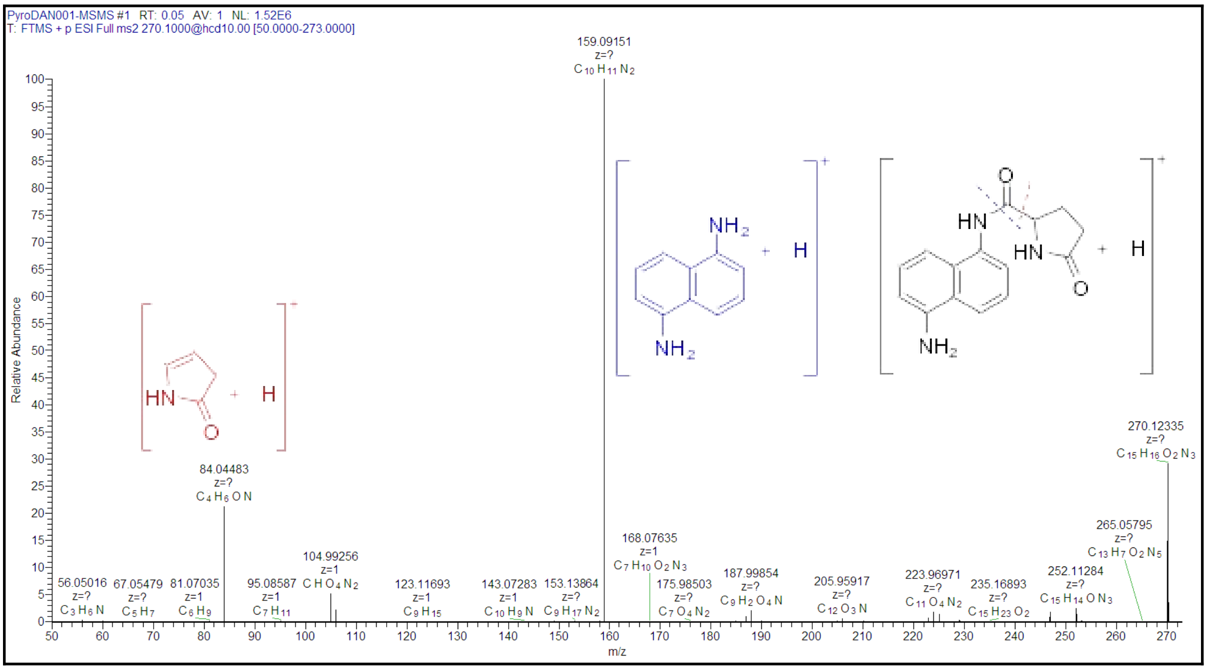

Figure S10. Higher-energy Collisional Dissociation (HCD) HiRes MS-MS (ESI+) spectrum of PyroDAN

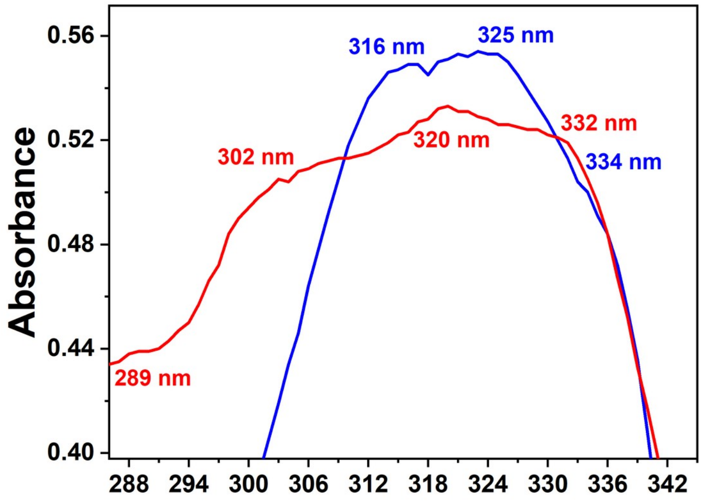

Figure S11. Expansion of the UV-Vis of DAN (blue line) and PyroDAN (red line) in water for the range 286-345 nm.

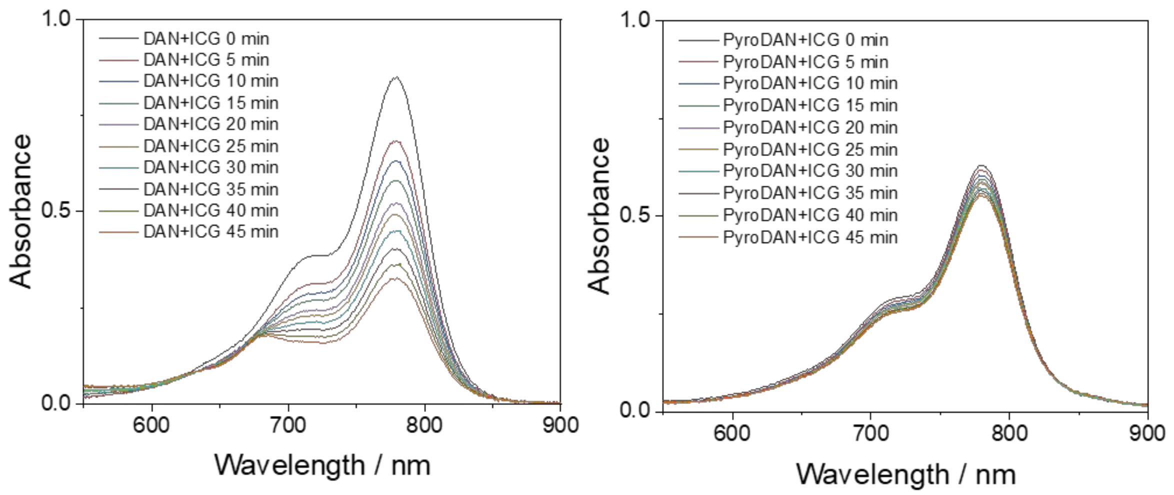

Figure S12. UV-Vis Absorption Spectra of ICG+DAN (**a**) and ICG + PyroDAN as a fucntion of time (**b**).

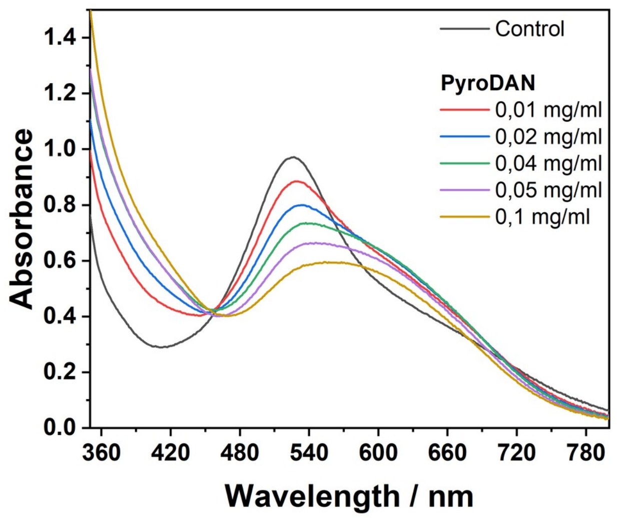

Figure S13. UV-Vis Absorption Spectra of DPPH with several concentration of PyroDAN ranging from 0.01 to 0.1 mg/ml.

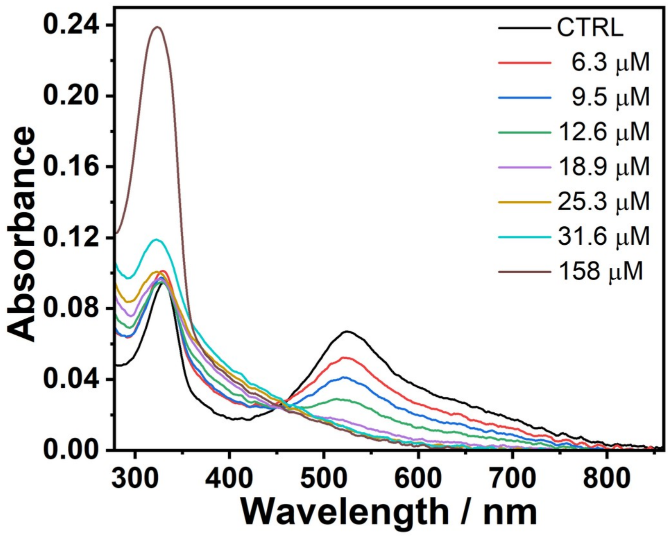

Figure S14. UV-Vis Absorption Spectra of DPPH with concentration of DAN ranging from 6.3 to 158 μM.
